# Demographic and clinical characteristics of dogs with centroblastic lymphoma

**DOI:** 10.14202/vetworld.2021.49-55

**Published:** 2021-01-07

**Authors:** Katarzyna Kliczkowska-Klarowicz, Dariusz Jagielski, Michał Czopowicz, Rafał A. Sapierzyński

**Affiliations:** 1 Department of Pathology and Veterinary Diagnostics, Division of Pathology, Institute of Veterinary Medicine, Warsaw University of Life Sciences (SGGW), Nowoursynowska 159c, 02-776 Warsaw, Poland; 2 Białobrzeska Veterinary Surgery in Warsaw, Poland; 3 Division of Veterinary Epidemiology and Economics, Institute of Veterinary Medicine, Warsaw University of Life Sciences (SGGW), Nowoursynowska 159c, 02-776 Warsaw, Poland

**Keywords:** Bernese mountain dogs, clinical stage, cytology, epidemiology, fine-needle biopsy, Golden Retrievers, immunophenotype, lymphoma, Rottweilers

## Abstract

**Background and Aim:**

Centroblastic lymphoma (CBL) is the most common morphological type of lymphoma found in dogs; it is usually identified through cytology in veterinary clinical practice. This study aimed to identify the demographic and clinical characteristics of dogs with CBL that was diagnosed with cytology and immunocytochemistry.

**Materials and Methods:**

Dogs with a suspicion of lymphoma were diagnosed by cytology supported by immunocytochemistry with the use of the updated Kiel classification adapted for dogs. During the analyzed time period, 336 lymphomas were diagnosed in dogs, including 171 cases of CBL. Epidemiological and clinical data from the dogs with CBL were provisionally collected.

**Results:**

The epidemiology analysis revealed an increased risk of CBL in Rottweilers, golden retrievers, and Bernese mountain dogs. At admission, most of the dogs displayed generalized lymphadenopathy with spleen and liver enlargement. The most common hematological abnormality was leukocytosis due to neutrophilia. The most common biochemical abnormality was elevated alanine aminotransferase and alkaline phosphatase activities and selective hypoproteinemia due to hypoalbuminemia.

**Conclusion:**

Rottweilers, Bernese mountain dogs, and golden retrievers appear to be overrepresented among dogs with CBL. CBL is usually diagnosed at an advanced clinical stage according to the World Health Organization; however, it is usually accompanied by only minor hematological and biochemical abnormalities.

## Introduction

Centroblastic lymphoma (CBL) is the most common type of lymphoma found in dogs. It accounts for more than half of all lymphoid neoplasms and 60-80% of B-cell lymphomas [1-9]. Despite its prevalence, there is a lack of comprehensive data concerning the epidemiology, clinical picture, and clinical pathology in dogs with CBL. More data have been reported in studies on diffuse large B-cell lymphoma (DLBCL), which can be considered a histological counterpart of CBL because it accounts for roughly 80% of all CBL cases [5,8,10].

The median age of dogs with CBL or DLBCL is 8 years [5,10-12]. The breed predisposition for CBL and DLBCL differs between studies. In a study by Davies *et al*. [11], Jack Russell terriers, golden retrievers, beagles, West Highland white terriers, and flat-coated retrievers were significantly more frequently affected by CBL. According to the previous studies, the most frequently described breeds with DLBCL are golden retrievers, Labrador Retrievers, Rottweilers, Doberman Pinschers, Bernese mountain dogs, and German Shepherds [10,12,13]. There does not appear to be any gender predisposition to CBL in dogs [5].

Dogs with CBL or DLBCL are diagnosed at all clinical stages of the disease, according to the World Health Organization (WHO); however, diagnoses at Stages III and IV with or without the presence of general signs are most common [4,9-12]. The most common hematological abnormalities are anemia, thrombocytopenia, leukocytosis, and lymphocytosis or lymphopenia [11,14,15]. The increased activities of alanine aminotransferase (ALT), alkaline phosphatase (ALP), lactate dehydrogenase, and hyperbilirubinemia are also frequently observed [11,14].

Histological examination is undoubtedly a diagnostic gold standard in oncology; however, the mainstay for diagnosing lymphadenopathy in dogs during routine veterinary practice remains cytology, which is performed on material collected through a fine-needle biopsy. Given the prevalence of CBL in dogs, identifying the epidemiological characteristics and providing clinical data (including the clinical stage of ­disease and blood check-up results) from a large number of dogs with cytologically diagnosed CBL may be of significant clinical importance.

This study aimed to identify the demographic and clinical characteristics of dogs with CBL.

## Materials and Methods

### Ethical approval

All data were obtained during the necessary diagnostic procedures; therefore, no ethics committee approval was necessary.

### Study location, period, population, and sample collection

This retrospective study was conducted on dogs that presented to two large veterinary clinics in Warsaw, Poland, during the years 2009-2016. During this period, demographic data, including sex, breed, and age of all dogs with cytologically diagnosed lymphoma were provisionally collected. Samples for cytological examination were obtained by fine-needle aspiration or fine-needle non-aspiration biopsy from enlarged lymph nodes or from other sites, including internal organs (liver and spleen), abnormal masses located on the skin and in the body cavities (mediastinal tumors, abdominal cavity tumors, and nasal cavity tumors), bone marrow and fluids from serous cavities, and peripheral blood. At least three samples were collected from at least two enlarged lymph nodes in cases of systemic lymphadenomegaly. Finally, only dogs in which a cytological diagnosis of CBL was established definitively by two clinical pathologists (KKK and RS) and in which the immunophenotype of lymphoma was confirmed by immunocytochemistry were enrolled in this study.

### Cytological examination

For the routine examinations, at least three smears of each aspirate were dried, fixed in 70% methanol, stained with Giemsa solution (Analab®) that was prepared immediately before staining according to the manufacturer’s instructions, and examined by light microscopy. For the immunocytochemical assays, smears from each dog were dried, fixed in acetone at 4°C for 5-10 min, and stained immediately or stored at −20°C. Immunocytochemical staining was performed according to the methods of Caniatti *et al*. [16] and Sapierzyński [17] using commercially available antibodies (Dako®, Denmark) for the pan-T-lymphocyte marker CD3 (FLEX, Polyclonal Rabbit ­Anti-Human, Dako, ready-to-use) and the B-cell antigen receptor complex CD79a (FLEX, Monoclonal Mouse Anti-Human, Dako, clone JCB117, ready-to-use). Two smears from each dog were stained using both antibodies. The expression intensity of the examined CD antigens in the cytological preparation was determined through light microscopy, and the result was considered positive if at least 80% of the lymphomatous cells displayed a strong cytoplasmic reaction. Negative controls were processed in the same way with a buffer solution instead of primary antibodies. The positive controls for CD3 and CD79a were cellular samples collected from impression smears of canine hyperplastic lymph nodes.

All cases were immunophenotyped, and those with CD79 receptor expression (immunophenotype B) were classified according to the updated Kiel classification adapted for canine lymphoma as previously described independently by two groups of pathologists [3,4,18]. The following features were determined: The size and shape of cells; cytoplasm volume and intensity of cytoplasm staining; size and shape of nuclei; the position of the nucleus in a cell; size, distinctness, number, and positioning of nucleoli; and the appearance of nuclear chromatin. Among the samples with a B-cell immunophenotype, those which consisted of a monotonous population of blastic lymphocytes with round nuclei and several prominent nucleoli located in the margin, those with scant, basophilic cytoplasm, and those with no more than 20% of medium macronucleolated cells or immunoblasts were classified as CBL [3,4,6,9].

Cell lines were not validated because cell lines were not used during this study.

### Clinical characteristics

The following clinical data were collected from the dogs enrolled in this study: Clinical examination (including clinical signs that were present during examination and abnormalities in the appearance or behavior reported by the owner), the results of routine blood cell count and serum biochemistry analyses (total protein, albumin, bilirubin, urea and creatinine concentrations, calcium concentration corrected for total protein or albumin concentration, ALT, aspartate aminotransferase, and ALP activity), and the presence of spleen or liver enlargement, and clinical stage of the disease according to the WHO. The hematological examination was performed using an automated hematology analyzer (scil Wet abc, HORIBA, Poland), and serum biochemistry analyses were performed using the chemistry analyzer IDEXX Catalyst One^®^ (IDEXX Laboratories, USA).

### Statistical analysis

Numerical variables were described as the median, interquartile range (IQR), and range and were compared between groups using the Mann–Whitney U-test. Categorical variables were presented as the count and percentage and were analyzed with either Pearson’s Chi-square test or Fisher’s exact test (depending on the expected values of the variable distribution). The Wilson score method was used to determine the 95% confidence intervals (CIs) for proportions [19]. Breed predisposition to CBL was investigated with a case-control approach by calculating the odds ratios (ORs). The theoretical distribution of dog breeds was developed to establish a control group, as previously described by Jankowska *et al*. [20]. Briefly, databases of two round-the-clock Polish general veterinary practices for the years 2009-2013 (50,000 different virtual dog patients) were used. Then, the counts necessary to determine the ORs were computed for a reference number of 10,000 dogs. The proportion of dogs of a certain breed diagnosed with CBL was compared with the proportion of dogs of the same breed that presented to the veterinary clinics for any other reason; these data were used to calculate the crude OR. The same procedure was repeated for each breed represented in the study by at least five dogs. The significance level (a) was set at 0.05. ORs with their 95% CIs were calculated using EpiTools [21]. All other analyses were performed in TIBCO Statistica 13.3.0 (TIBCO Software Inc., Palo Alto, CA).

## Results

During the study period, 336 dogs were diagnosed with lymphoma, of which 171 (50.9%) met the eligibility criteria and were included for further analysis. Males (n=89, 52.1%) and females (n=82, 47.9%) were virtually equally represented, and most of the dogs were neutered (75% of males and 76% of females). The dogs ranged in age from 1.5 to 19 years with a median age of 8 years (IQR, 6-11 years); age did not differ significantly between sexes (p=0.494). Forty-eight dogs were mongrels (28.1%), and the rest belonged to 40 breeds, of which the following were represented by at least five individuals: Rottweilers (n=11, 6.4%), German shepherds (n=10, 5.8%), Bernese Mountain dogs (n=9, 5.3%), Labrador retrievers (n=8, 4.7%), golden retrievers (n=7, 4.1%), and dachshunds, Yorkshire terriers, and miniature schnauzers (each n=5, 2.9%). Three breeds (Rottweilers, Bernese mountain dogs, and golden retrievers) were significantly overrepresented and two breeds (dachshunds and Yorkshire terriers) were significantly under-represented among dogs with CBL (Table-1).

**Table-1:** Distribution of centroblastic lymphoma in dog breeds represented in the study population by at least five individuals.

Breed	Polish theoretical distribution of 10,000 dogs n (%)	Dogs affected with centroblastic lymphoma (n=171) n (%)	OR	CI 95%	p-value
Crossbreed	3096 (31.0)	48 (28.1)	0.9	0.6, 1.2	0.418
Rottweiler^a^	146 (1.5)	11 (6.4)	4.6	2.5, 8.7	<0.001*
German Shepherd	727 (7.3)	10 (5.8)	0.8	0.4, 1.5	0.478
Bernese mountain dog^a^	41 (0.4)	9 (5.3)	13.5	6.4, 28.2	<0.001*
Labrador Retriever	295 (2.9)	8 (4.7)	1.6	0.8, 3.3	0.187
Golden Retriever^a^	152 (1.5)	7 (4.1)	2.8	1.3, 6.0	0.020*
Miniature Schnauzer	248 (2.5)	5 (2.9)	1.2	0.5, 2.9	0.619
Dachshund^b^	988 (9.9)	5 (2.9)	0.3	0.1, 0.7	0.002*
Yorkshire terrier^b^	727 (7.3)	5 (2.9)	0.4	0.2, 0.9	0.024*

a Breeds significantly over-represented among dogs affected by centroblastic lymphoma;

b Breeds significantly under-represented among dogs affected by centroblastic lymphoma. OR=Odds ratio, CIs=Confidence intervals,

* Statistically significant.

### Clinical data

**Figure-1:**
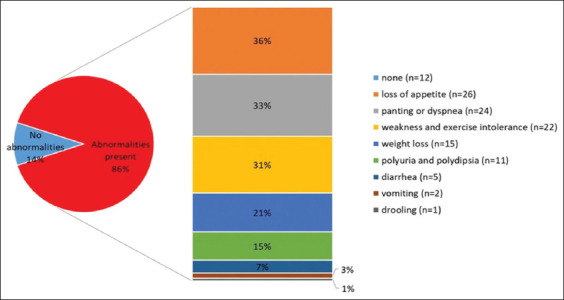
Abnormalities in the appearance and behavior observed by the owners in 86 dogs with centroblastic lymphoma.

Detailed clinical data were available for 84 dogs with CBL. The owners of 12 dogs (14%) did not notice any abnormalities, and CBL was found only incidentally during routine clinical examinations before vaccination or elective surgery or by detecting lymphadenomegaly and less often splenomegaly. Those abnormalities were the most common in dogs with CBL and were present in 80 (95%) and 59 (70%) dogs, respectively. Fever was noted in 26 of the 84 dogs (31%). Subjective clinical signs were observed by the owners in 72 dogs (86%) (Figure-1). According to the WHO clinical staging scheme, 56 dogs (67%) were classified as Stage IV (of which 46 dogs were IVB), 25 dogs were classified as Stage III (30%; 19 dogs were IIIB), and three dogs were classified as Stage V (3%; all were VB).

### Hematology and biochemistry

Detailed hematological results were available for 69 dogs with CBL (Table-2). The most common abnormality was leukocytosis resulting from neutrophilia with band neutrophils. Anemia and thrombocytopenia (usually mild and less commonly moderate) were observed in 31% and 43% of dogs, respectively. The most common biochemical abnormalities were aminotransferase and ALP activity elevation and decreased total protein and albumin concentrations. Azotemia and hypercalcemia were rare (Table-3).

**Table-2:** Hematological analysis of dogs with centroblastic lymphoma.

Parameter (Reference range^a^)	n	Median, IQR, Range	Elevated n (%)	Lowered n (%)
White blood cell count (6-16 G/L)	69	10.8, 8.3-19.1 (2.6-41.4)	22 (32)	6 (9)
Segmented Neutrophil count (3.6-12 G/L)	69	8.1, 5.6-11.8 (0.9-31.9)	17 (25)	4 (6)
Band neutrophil count (≤0.36 G/L)	68	0.1, 0-0.5 (0-4.5)	21 (31)	-
Lymphocyte count (0.7-3.6 G/L)	67	1.6, 1.0-3.7 (0-15.7)	17 (25)	7 (10)
Eosinophil count (0.1-1.2 G/L)	51	0.1, 0-0.3 (0-1.6)	4 (8)	-
Monocyte count (0-1.2 G/L)	50	0, 0-0.4 (0-13.8)	9 (18)	-
Hematocrit (0.37-0.55)	68	0.40, 0.37-0.46 (0.22-0.61)	3 (4)	17 (25)
Hemoglobin (7.5-11.2 mmol/L)	69	8.5, 7.7-9.6 (4.4-13.4)	5 (7)	14 (20)
Red blood cell count (5.5-8.0 T/L)	68	5.9, 5.3-6.8 (2.8-9.2)	4 (6)	21 (31)
Platelet count (200-580 G/L)	69	204, 149-290 (46-747)	1 (1)	30 (43)

**Thrombocytopenia**		**n (%)**		

Mild (100-200)		22 (74)		
Moderate (50-100)		7 (23)		
Severe (<50)		1 (3)		

a Reference ranges of the laboratory in which samples were tested. IQR=Interquartile range

**Table-3:** Biochemical analysis of dogs with centroblastic lymphoma.

Parameter (Reference range^a^)	n	Median, IQR, Range	Elevated n (%)	Lowered n (%)
Calcium corrected^b^ (2.1-2.9 mmol/L)	53	2.6, 2.4-2.7 (2.1-3.0)	2 (4)	1 (2)
Total protein (55-75 g/L)	62	63, 58-69 (42-88)	3 (5)	11 (18)
Albumin (29-43 g/L)	55	31, 28-34 (13-48)	2 (4)	16 (29)
Alanine aminotransferase (<60 U/L)	62	50, 30-83 (15-540)	26 (42)	-
Aspartate aminotransferase (<45 U/L)	38	46, 31-59 (8-487)	9 (24)	-
Alkaline phosphatase (<155 U/L)	41	111, 45-256 (16-1921)	27 (66)	-
Bilirubin (<15.4 μmol/L)	42	5.1, 3.4-8.6 (1.7-179.6)	2 (5)	-
Urea (3.3-8.3 mmol/L)	67	5.3, 4.2-7.0 (1.7-37.8)	12 (18)	6 (9)
Creatinine (<150 μmol/L)	67	88.4, 79.6-106.1 (53.0-380.2)	2 (3)	-

a Reference ranges of the laboratory in which samples were tested;

b Ca corrected for albumin or total protein concentration. IQR=Interquartile range

## Discussion

Cytological examination is the most popular diagnostic test used to investigate the causes of general lymphadenopathy in dogs in veterinary practice. Cytology supported by immunocytochemistry allows pathologists to make a true diagnosis of lymphoma in most cases; therefore, it is usually accepted as an accurate and reliable diagnostic method in scientific studies [11,14,22-25]. The prognostic value of the Kiel classification scheme, which is widely used in the cytological examination of lymphoma in dogs, was proven by Ponce *et al*. [4]. In the USA, virtually 90% of veterinary surgeons (including certified oncologists) claim to use cytology for recognizing lymphoma in dogs with additional immunocytochemistry used in 75% of cases. Less than 30% of veterinary surgeons perform histopathological examinations on surgically removed lymph nodes [26].

In the authors’ opinion, it is important to report epidemiological and demographical data collected on large numbers of animals affected by certain diseases because these data can be used to create large epidemiological databases. Comparing data between affected animals or between certain diseases may improve our understanding of the pathogenesis of these diseases and identify potential groups for detecting genetic predispositions.

In our study, more than half of all cases of B-cell lymphoma were classified as CBL, which is consistent with the previous observations [1-3,5,6,8].

CBL is more common in middle-aged to old dogs, which is similar to humans in whom the median age for DLBCL falls between the sixth and seventh decades [27]. The median age of dogs with CBL (8 years) in our study was similar to the median ages of dogs with CB, DLBCL, and “large B-cell lymphoma” (LBCL) reported by other authors [5,6,10], B-cell lymphoma in the studies of Sӧzmen *et al*. [6], Marconato *et al*. [14], and lymphoma in general [7,20,28]. Given that CBL is the most common type of lymphoma, these results are not surprising.

In our study, Rottweilers, Bernese mountain dogs, and golden retrievers were overrepresented among dogs with CBL, which may indicate that they are predisposed to this disease. These breeds have also been overrepresented among dogs with DLBCL/LBCL in the studies by Childress *et al*. [10], Marconato *et al*. [13,14] (Rottweilers, golden retrievers), and Sierra Matiz *et al*. [12] (Rottweilers). Rottweilers, Bernese mountain dogs, and golden retrievers were also among the eight breeds presumably predisposed to lymphoma regardless of its type in a study by Jankowska *et al*. [20]. Given the high prevalence of CBL among lymphomas in dogs, a predisposition to lymphoma (regardless of the type) in the aforementioned breeds may actually reflect a predisposition to CBL. Moreover, in the study by Jankowska *et al*. [20], a decreased predisposition to lymphoma was noted in dachshunds and Yorkshire terriers, similar to this study for CBL. The overrepresentation of certain breeds among dogs with CBL may indicate the presence of genetic predispositions in these breeds and may be a subject of future research.

An important limitation of this study is the fact that conclusions regarding breed predisposition are based on a univariable analysis because the dogs with CBL were not directly compared with a control group composed of dogs without lymphoma. In fact, only the distribution of canine breeds observed in our study was compared with a theoretical canine breed distribution from Warsaw veterinary clinics. This theoretical canine breed distribution reflects the frequency with which a given breed is presented to the veterinary clinic at all. When a given breed shows up at the clinic with CBL significantly more frequently than it shows up at the clinic for other reasons, we can infer that this breed appears to be more likely to have CBL. Therefore, we prefer to use the term overrepresented among dogs with CBL rather than predisposed to CBL. True breed predisposition should have been investigated with a multivariable analysis, which controls for potential confounding factors. This was not, however, our main goal in this study.

Most of the dogs had a high clinical stage of disease (IV according to the WHO), and no dogs had Stage I or II disease, which is similar to previous observations [10,11,12]. However, over 80% of the dogs in our study displayed general clinical symptoms, while this proportion rarely exceeded 50% in other studies. This may be the result of the long time that generally elapses between the first medical consultation and the moment when definitive diagnosis is made in Poland.

Only a few dogs in our study had Stage V disease according to the WHO (the presence of neoplastic cells in the blood or bone marrow); conversely, this stage was described in 34% of dogs in a study by Childress *et al*. [10]. Stage V disease was recognized on the basis of blood smears alone, similar to the study by Davies *et al*. [11]; however, in a study by Childres *et al*. [10], all dogs with the suspicion of Stage V disease underwent a bone marrow biopsy. The lack of neoplastic cells in the blood does not mean that they are not present in the bone marrow [13]. Because thrombocytopenia, leukocytosis, and lymphocytosis were observed in roughly one-third of the dogs in our study, which may be indicative of massive bone marrow involvement [15], we suspect that the number of dogs with Stage V disease in our work was likely underestimated. Despite the fact that the involvement of bone marrow by lymphoma corresponds to a worse prognosis [29,30], bone marrow biopsy is still considered an invasive and life-threatening procedure and is performed in less than half of the patients with lymphoma [31].

Reports concerning hematological and clinical biochemistry results in dogs with CBL are sparse. Parameters are usually reported as the median without the percentage of dogs showing certain abnormalities. Anemia is a common abnormality in dogs with lymphoma, including CBL. It is frequently caused by hemolysis on immunological background or bone marrow involvement. The percentage of dogs with anemia in our study (31%) was similar to the results reported by other authors such as Marconato *et al*. [14] and Davies *et al*. [11]. Interestingly, in a study by Childress *et al*. [10], this value was only half that of the current study, as was the percentage of dogs with thrombocytopenia (22% vs. 43% in our study).

## Conclusion

Rottweilers, Bernese mountain dogs, and Golden Retrievers appear to be overrepresented among dogs with CBL. CBL is usually accompanied by only minor hematological and biochemical abnormalities.

## Authors’ Contributions

RAS and KK contributed in conceptualization and methodology. RAS, KK, and DJ collected the samples. DJ provided clinical data. RAS and KK conducted the laboratory examinations. MC performed data organization, software analysis, and visualization. KK and MC prepared original draft and editing with the supervision of RAS. All authors have read and agreed to the published version of the manuscript.
